# Morphological Determinants of Carbon Nanomaterial-Induced Amyloid Peptide Self-Assembly

**DOI:** 10.3389/fchem.2020.00160

**Published:** 2020-03-10

**Authors:** Yanting Xing, Yunxiang Sun, Bo Wang, Feng Ding

**Affiliations:** Department of Physics and Astronomy, Clemson University, Clemson, SC, United States

**Keywords:** amyloid peptide self-assembly, beta-sheet supercoil, carbon nanotube, graphene, discrete molecular dynamics

## Abstract

Hybridizing carbon nanomaterials (CNMs) with amyloid fibrils—the ordered nanostructures self-assembled by amyloidogenic peptides—has found promising applications in bionanotechology. Understanding fundamental interactions of CNMs with amyloid peptides and uncovering the determinants of their self-assembly structures and dynamics are, therefore, pivotal for enriching and enabling this novel class of hybrid nanomaterials. Here, we applied atomistic molecular dynamics simulations to investigate the self-assembly of two amyloid peptides—the amyloidogenic core residues 16-22 of amyloid-β (Aβ_16−22_) and the non-amyloid-β core of α-synuclein (NACore_68−78_)—on the surface of carbon nanotubes (CNT) with different sizes and chirality. Our computational results showed that with small radial CNTs, both types of peptides could form β-sheets wrapping around the nanotube surface into a supercoiled morphology. The angle between β-strands and nanotube axes in the supercoil structure depended mainly on the peptide sequence and CNT radius, but also weakly on the CNT chirality. Large radial CNTs and the extreme case of the flat graphene nanosheet, on the other hand, could nucleate amyloid fibrils perpendicular to the surface. Our results provided new insights of hybridizing CNMs with amyloid peptides and also offered a novel approach to manipulate the morphology of CNM-induced amyloid assembly by tuning the surface curvature, peptide sequence, and molecular ratio between peptides and available CNM surface area, which may be useful in engineering nanocomposites with high-order structures.

**Graphical Abstract:**
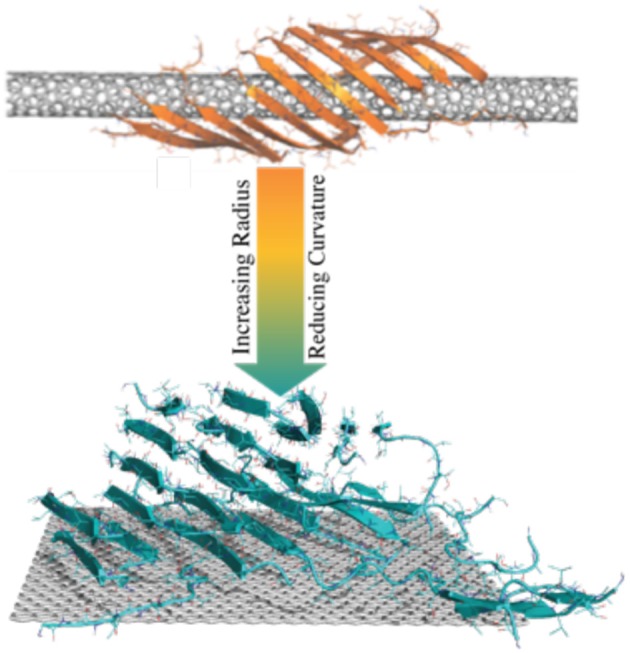
Amyloid peptides could form β-sheets wrapping around the surface of CNTs with small radius into a supercoil structure. As the radius increases, CNTs with small curvatures or flat graphene nanosheets could nucleate amyloid fibrils perpendicular to the surface.

## Introduction

Carbon nanomaterials (CNMs) including fullerene (Krusic et al., 1991), nanotube (Peigney et al., 2001; Ge et al., 2018), and graphene (Allen et al., 2010; Qian et al., 2010) have been explored for numerous applications in energy, computing, environment, and medicine. CNMs are also widely used as building blocks for hybrid nanocomposites (Allen et al., 2010; Qian et al., 2010). To enable their broad applications in bionanotechnology, CNMs can be hybridized with biomacromolecules (Chan, 2006) to improve their aqueous solubility (Ke and Qiao, 2007), lower their cellular toxicity (Liu et al., 2010) for tissue engineering and drug delivery (Wang et al., 2011; Sanchez et al., 2012; Lee et al., 2009), and harness novel functionalities (Sun et al., 2016). Among various biomacromolecules, proteins are highly advantageous due to their capability of conjugation with CNMs (Li et al., 2017; Hauser et al., 2014), biological compatibility (Buchanan et al., 2014; Jacob et al., 2016), wide availability (Luo et al., 2016), great chemical and structural varieties (Nguyen et al., 2016). In particular, amyloid nanofibers self-assembled by proteins and polypeptides have drawn many attentions due to their unique nanostructures (Cherny and Gazit, 2008; Liu et al., 2011) and high designability in the amino acid sequence space. Traditionally associated with a long list of amyloid diseases (e.g., Alzheimer's disease Childers et al., 2012; Liang et al., 2014, Parkinson's disease Rodriguez et al., 2015, and type-2 diabetes Chun Ke et al., 2017) and also functional (Fowler et al., 2006, 2007) roles in biology, amyloid fibrils have been discovered as building blocks for bionanotechnology (Knowles and Mezzenga, 2016) and used to develop hybrid CNMs for novel applications. Conjugating β-lactoglobulin amyloid fibrils with sulfonated multi-walled carbon nanotubes (MWCNTs) via both covalent cross-link and physical π-π interactions, Li and Mezzenga (Li and Mezzenga, 2012) developed biocompatible, pH-responsive, and fully fibrous hydrogels far below the gelling concentration of amyloid nanofibers. Li et al. (2012) hybridized β-lactoglobulin amyloid nanofibrils with graphene oxide to fabricate biodegradable nanocomposites with shape-memory and enzymatic sensing properties. Javed et al. (2018) coated CNTs with fragmented β-lactoglobulin amyloid nanofibrils and demonstrated both *in vitro* and *in vivo* that the hybrid CNTs could mitigate the aggregation-associated cytotoxicity of both islet amyloid polypeptide (IAPP, a.k.a. amylin) in type-2 diabetes and amyloid-β (Aβ) in Alzheimer's disease.

In addition to pre-formed amyloid fibrils as the functionalization agents, the adsorption and self-assembly of amyloid polypeptides onto the surface of CNMs were also studied both *in vitro* and *in silico*. Using *in situ* atomic force microscopy (AFM), Kowalewski and Holtzman showed that Aβ_1−42_, when deposited on graphite, formed ordered elongated β-sheets with extended conformations. Combining AFM and molecular dynamics (MD) simulations, Terán Arce et al. (2011) suggested that a strong interaction between Aβ_17−42_ and highly ordered pyrolytic graphite (HOPG) forced the formation of amyloid filaments following graphite's hexagonal lattice symmetry, which was validated in simulations with pre-assembled Aβ_17−42_ filament on HOPG resulting into a stable U-shaped β-turn-β structure along the graphene surface. Lin et al. (2016) observed the rapid adsorption of Aβ_1−40_ on single-walled carbon nanotubes (SWCNTs) surfaces and subsequent inhibition of amyloid aggregation into toxic oligomers and mature fibrils using both biophysical characterization and coarse-grained MD simulations. While many of these works offered useful insights to the complex interactions between specific types of amyloid peptides and CNMs, the parameter space of CNMs (e.g., dimension, chirality, and curvature) and also the sequence space of amyloid peptides are very large and thus the physicochemical determinants of amyloid peptide-CNM interactions including the assembly structures and dynamics are still unknown. Such a knowledge gap hinders the development of this novel class of hybrid nanomaterials and limits their broad applications.

Here, we attempt to fill the knowledge gap by investigating the self-assembly of two types of amyloid peptides with different sequences and lengths on the surface of CNTs with different chirality and radii as well as flat graphene nanosheets using atomistic discrete molecular dynamics (DMD) simulations. DMD is a rapid and predictive MD algorithm, widely used to study proteins (Pilkington et al., 2017), amyloid aggregation (Ge et al., 2018), nanoparticles (NPs) and Nano-Bio interactions (Wang et al., 2018). Different CNTs and the graphene nanosheet were selected to sample the parameters of CNMs, including dimension, chirality, and curvature. We chose the 7-residue amyloidogenic core of Aβ (^16^KLVFFAE^22^, Aβ_16−22_) in Alzheimer's disease and the 11-residue non-amyloid-β core of α-synuclein (^68^GAVVTGVTAVA^78^, NACore_68−78_) in Parkinson's disease as the model peptides in our simulations. In the absence of CNMs, both peptides could spontaneously form amyloid fibrils *in vitro* (Potter et al., 2010) and *in silico* (Sun et al., 2018). From DMD simulations, we found that both peptides bound strongly to CNTs of different structural parameters. For CNTs with small radii (i.e., large curvatures), the peptides could form β-sheets coiled along the CNT surface where side-chains on one side of the β-sheets interacted with the NP. The supercoil morphology such as the angle between β-strand and nanotube axis depended strongly on the peptide sequence and CNT radius, but also weakly on the CNT chirality. With increased CNT radius, the peptide aggregates became increasingly disordered with reduced β-sheet content. The peptides preferred to bind the large radii CNTs with increased contacts to the NPs and decreased inter-peptide interactions. These peptides not forming β-sheets tended to bind the CNT in a *straddle* conformation where all side-chains were in contact with the flattened NP surface. Interestingly, such a surface-bound peptide conformation on the relatively flat CNM surface had the backbone hydrogen bond donors and acceptors exposed and sticking out of the NP surface. With excessive peptides compared to the accessible CNM surface, we postulated that incoming peptides might form β-sheets with those backbone-exposed peptides on the NP surface and nucleate the growth of amyloid fibrils perpendicular to the NP surface, which was confirmed by our DMD simulations of a graphene nanosheet with excessive peptides. Hence, our results not only provided new insights of hybridizing CNMs with amyloid peptides and also offered a novel approach to control the morphology of CNM-induced amyloid assembly by tuning the surface curvature, peptide sequence, and molecular ratio between peptides and available CNM surface area.

## Materials and Methods

### Discrete Molecular Dynamics (DMD) Simulations

DMD is a special type of molecular dynamics algorithm where conventional continuous inter-atomic interaction potential functions are replaced by optimized step functions (Rapaport; Allen and Tildesley, 2017). A comprehensive description of the atomistic DMD algorithm was published elsewhere (Ding et al., 2008; Ding and Dokholyan, 2012). In brief, the united-atom model represents all molecules where all heavy atoms and polar hydrogen atoms are explicitly modeled and an implicit solvent model was adopted to capture the solvent effect. Inter-atomic interactions included van der Waals, solvation, electrostatic and hydrogen bonds. The solvation energy was estimated according to the Lazaridis-Karplus implicit solvent model, EEF1 (Lazaridis and Karplus, 2000). The distance- and angular-dependent hydrogen bond interaction was modeled using a reaction-like algorithm (Ding et al., 2003). Screened electrostatic interactions between formal charges were computed by the Debye-Hückel approximation, where a Debye length of 1 nm was used by assuming a water dielectric constant of 80 and a monovalent electrolyte concentration of 0.1 M. The Anderson's thermostat (Andersen, 1980) was used to maintain constant temperature. The DMD program is available via Molecule in Action, LLC (http://www.moleculesinaction.com/).

### Simulation System Setup and Analysis

The initial structural coordinates for NACore_68−78_ and Aβ_16−22_ were obtained from protein data bank (PDB). CNTs and graphene structures were generated from visual molecular dynamics (VMD) (Humphrey et al., 1996). To capture the chirality effect of CNT on peptide self-assembly, zigzag (*n* = 10, *m* = 0), armchair-like (*n* = 6, *m* = 5), and chiral (*n* = 8, *m* = 3) CNTs with the diameter of ~7.6 Å were selected. For the effect of varying CNT radii, a battery of chiral CNTs (an angle of ~15°) with radii (R) and height (H) ranging from R_0_ ~2.0 Å, H_0_ ~120.0 Å (*n*=5, *m*=2); R_1_ ~3.8 Å, H_1_ ~100.0 Å (*n* = 8, *m* = 3); R_2_ ~7.6 Å, H_2_ ~76.9 Å (*n* = 16, *m* = 6), R_3_ ~10.7 Å, H_3_ ~59.6 Å (*n* = 22, *m* = 8); R_4_ ~14.2 Å, H_4_ ~47.4 Å (*n* = 29, *m* = 11), to R_5_ ~28.2 Å, H_5_ ~47.4 Å (*n* = 58, *m* = 22) were used such that the total surface area was approximately the same. For each of CNTs with different radii, 20 peptides were simulated to study their self-assembly structures and dynamics in the presence of one CNT. To maintain approximately the same peptide concentration, simulation box with 100 × 100 × 130 Å^3^, 100 × 100 × 130 Å^3^, 122 × 122 × 81 Å^3^, 144 × 144 × 61 Å^3^, 161 × 161 × 49 Å^3^, 151 × 151 × 49 Å^3^, were used for CNTs with radius equal to R_0_, R_1_, R_2_, R_3_, R_4_, and R_5_, correspondingly. The corresponding peptide concentration was ~25.6 mM. Compared to typical experimental studies of amyloid aggregation, higher peptide concentrations are usually used in MD simulations to reduce computational costs spent on calculating diffusions. Coarse-grained simulations showed that peptide concentrations could affect the aggregation kinetics (Radic et al., 2015). On the other hand, a previous computational aggregation study of insulation fragments at different concentrations (e.g., 3.3, 8.3, 16.6, and 83 mM) using all-atom MD simulations with explicit solvent (Matthes et al., 2012) suggested the aggregation structures was independent of the initial peptide concentration. The simulation box dimension for peptide aggregation in the presence of graphene was 61 × 61 × 70 Å^3^. For each molecular system, 50 independent simulations with different initial inter-molecular distances and orientations were performed at 300 K. Each simulation lasted 400 ns so that an accumulative 10 μs total simulation were done for each molecular system.

We used the dictionary secondary structure of protein (DSSP) method in our analysis of different secondary structure contents (Kabsch and Sander, 1983). An atomic contact was defined using an inter-atomic distance cutoff of 6.0 Å. Inter-chain peptide interactions were analyzed by the residue-residue contact frequency. The two-dimensional potential of mean force (2D PMF) for peptide-CNT binding was calculated according to *PMF* = - *k*_*B*_*T ln P(n*_*A*_*, n*_*B*_*)*, where *k*_*B*_ was the Boltzmann constant, *T* corresponded to the simulation temperature of 300 K, and *P*(*n*_*A*_, *n*_*B*_) was the probability of the number of residues binding CNT (*n*_*A*_) and the number of residues in coil or β-sheet conformation (*n*_*B*_).

## Results and Discussion

We investigated the effects of CNT chirality and curvature on amyloid peptide self-assembly in DMD simulations. We performed aggregation simulations of 20 Aβ_16−22_ or NACore_68−78_ peptides in the presence of different chiral CNTs with a similar diameter of ~7.6 Å (i.e., zigzag, chiral, and armchair) and also chiral CNTs with different radii ranging from 2.0 to 28.2 Å (R_0_ ~2.0 Å, R_1_ ~3.8 Å, R_2_ ~7.6 Å, R_3_ ~10.7 Å, R_4_ ~14.2 Å, and R_5_ ~28.2 Å). In these simulations, we kept the CNT surface area constant by adjusting their heights while maintaining the total volume to ensure the same peptide concentration (Materials and Methods). For each molecular system, 50 independent simulations with the simulation time of 400 ns were performed to ensure sufficient sampling. In each independent simulation, 20 peptides were initially placed randomly around the CNT with different initial inter-molecular distances and orientations. All simulations were performed at 300 K with the CNT kept static and peptides free to diffuse, undergo conformational changes, and also interact with other peptides and CNT.

### The Effect of CNT Chirality on Amyloid Peptide Self-Assembly

**Figure 1:**
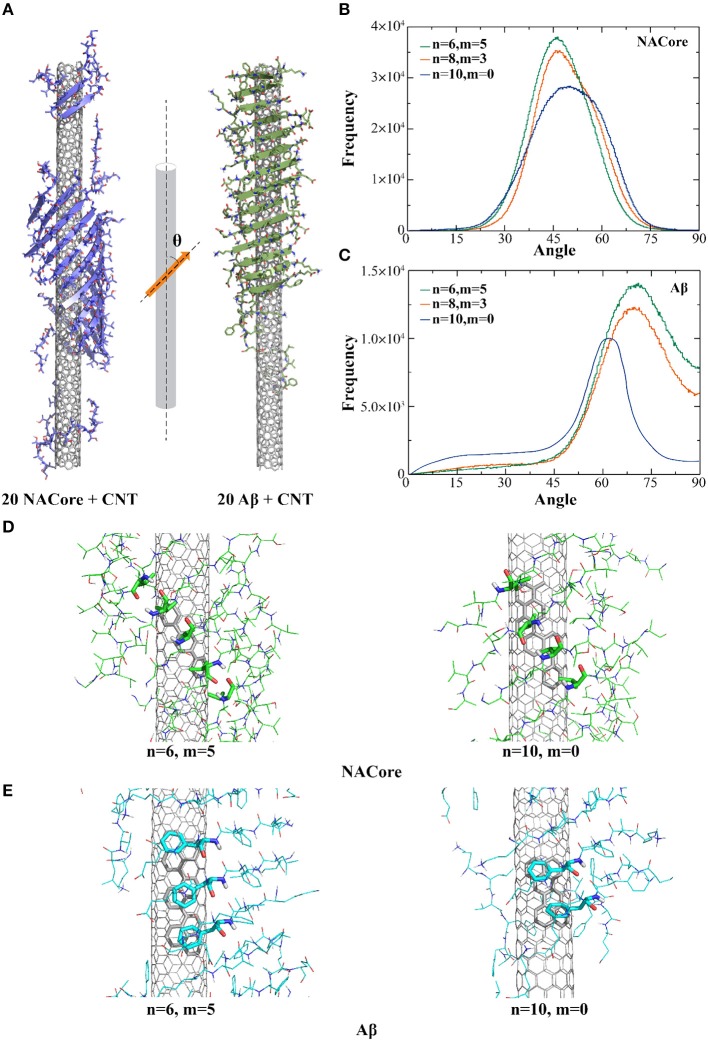
Self-assembly of amyloid peptides in the presence of different chiral CNTs. **(A)** Typical snapshot structures of peptides forming β-sheet supercoils on the surface of CNTs, where NACore (purple) and Aβ (green) were shown in cartoon representation. The distribution of angles between β-strands and CNT axis were computed for the self-assemblies of NACore **(B)** or Aβ **(C)** in DMD simulations. Representative binding configurations of NACore **(D)** and Aβ **(E)** with armchair-like (*n* = 6, *m* = 5) and chiral (*n* = 10, *m* = 0) CNTs, where hydrophobic (Valine residues in NACore) or aromatic (Phenylalanine residues in Aβ) interactions with CNTs were highlighted as sticks.

In the presence of CNTs having similar radii of ~7.6 Å but with different chirality, both NACore_68−78_ and Aβ_16−22_ fragments could self-assemble into β-sheet supercoils wrapping around CNT surfaces (Figure 1A). Using the last 200 ns of all independent simulation trajectories where steady states were achieved (e.g., representative trajectories shown in Figure S1), we computed the secondary structure contents of NACore_68−78_ and Aβ_16−22_ in the presence of CNT (Figure S2). All the amyloid peptides mainly adopted either β-sheet or random-coil structures. The inter-peptide contact frequency maps among side-chains or main-chains of individual residues (Figure S3) suggested that Aβ_16−22_ fragments preferred to form in-registered anti-parallel β-sheets (Figure S3B) due to opposite charges of K16 and E22 at both termini, consistent with our previous simulations (Sun et al., 2017). NACore_68−78_ formed both in-registered anti-parallel and parallel β-sheets (Figure S3A), likely due to its relatively even positioning of hydrophobic valine along the sequence.

To characterize the morphology of self-assembled β-sheet supercoils on CNT surfaces, we computed angles (θ) between CNT axis and the local β-strand directions at each β-strand residue, where the vector connecting Cα atoms of two neighbor residues denoted the local β-strand axis (Figure 1A). Using the last 200 ns of all trajectories, the histograms of the computed θ angles (Figures 1B,C) suggested that both NACore_68−78_ and Aβ_16−22_ had higher propensity to form β-sheets on the surface of chiral CNTs [e.g., CNTs (8, 3) and (6, 5) in Figures 1B,C] with higher peaks that than the zigzag CNT (10, 0). With CNTs of different chirality, orientation of the 11-residue NACore_68−78_ β-sheets had peaks at θ ~ 45°-50°, while 7-residue Aβ_16−22_ β-sheets had peaks at θ ~ 62°-72°. In the case of Aβ_16−22_, there were five consecutively hydrophobic residues, ^17^LVFFA^21^, which induced a strong hydrophobic interaction with CNT, especially the π-π stacking between aromatic groups of Phenylalanines (F) and CNT with highest binding probabilities as shown in Figure S4. To maximize the contact with CNT by burying the central hydrophobic FF region, the peptides in a rigid β-sheet conformation tended to align perpendicularly to the CNT axis with a relatively large θ angle (Figure 1A to the right). The resulted β-sheet supercoils had larger pitch length. On the other hand, NACore_68−78_ had multiple hydrophobic valines (V) positioned approximately evenly along the sequence with high binding probabilities the CNT (Figure S4). To optimize the binding of each peptide in the β-sheet with the CNT, NACore_68−78_ preferred to align parallel to CNT with a smaller θ value and the formed β-sheet supercoils had a shorter pitch length (Figure 1A on the left). Representative binding configurations of NAcore and Aβ on the zigzag and armchair SWCNTs were shown in Figures 1D,E. By highlighting interactions between hydrophobic (Valine in NACore) or aromatic (Phenylalanine in Aβ) residues aligned along β-sheets and contacting atoms of CNTs, the results suggested that armchair or chiral SWCNTs could better interact with these aligned hydrophobic or aromatic residues in β-sheets tilted for maximizing contacts with these highly curved SWCNT. In the case of zigzag CNTs, the competition between aligning these hydrophobic or aromatic residues along the CNT axis (due to interactions with carbon atoms inter-connected by bonds parallel to the zigzag CNT axis, Figures 1D,E to the right) and tilting β-sheets for increased contact with CNT resulted into reduced binding. Therefore, both Aβ_16−22_ and NACore_68−78_ can self-assemble into in-register β-sheet supercoils onto the surface of CNTs with radii ~7.6 Å with a weak preference to armchair and chiral CNTs. Distributions of hydrophobic and aromatic residues along the peptide sequence determines the morphology of the supercoils, such as the pitch lengths.

### The Effect of CNT Radii and Thus Surface Curvature on Amyloid Peptide Self-Assembly

**Figure 2:**
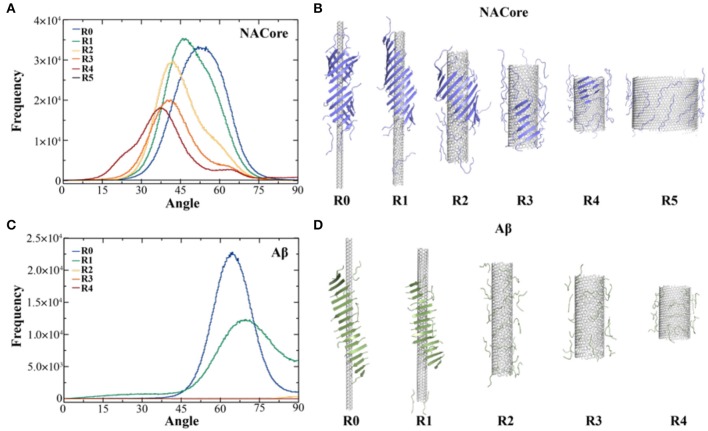
Self-assembly of amyloid peptides in the presence of CNT of different radii. **(A)** The distribution of angles between CNT axis and β-strands for the assembly of 20 NACore with CNT of different radii, ranging from R0 ~2.0 Å, R1 ~3.8 Å, R2 ~7.6 Å, R3~10.7 Å, R4~14.2 Å, to R5~28.2 Å. **(B)** The corresponding typical snapshots for CNT of different radii were shown. Similar results for Aβ were shown in panels **(C,D)**.

Aggregation simulations of both peptides in the presence of chiral CNTs with various radii were performed to examine the curvature effect of CNTs on peptide self-assembly. Using the equilibrated simulation trajectories, the distribution of θ angles between β-strands and CNT axis was computed for different molecular systems (Figure 2). As shown in Figure 2A, the θ angle distributions shifted from 50° to 37° as the radius of CNT increased from ~2.0 to ~14.2 Å (Figure 2A). Meanwhile, the probability of forming β-sheets decreased with increasing CNT radii as indicated the reduced total area under the curve and also illustrated by typical snapshots from simulations (Figure 2B). There was no obvious β-sheet formed when the radius increased to ~28.2 Å, and the peptides adsorbed to the CNT surface were mostly in coil confirmations. It is interesting to notice that NACore_68−78_ self-assembled into barrel-like structures around the narrowest CNT (e.g., R0 ~2.0 Å, Figure 2B to the left). Such barrel-like structures in the presence of ultra-thin CNTs were also observed using all-atom MD simulations with explicit solvent (Fu et al., 2009). Similarly, as the radius of CNT increased, the propensity of Aβ_16−22_ forming β-sheets also significantly decreased (Figures 2C,D). The β-sheet rich aggregates were only observed in small radii CNTs with R0 ~2.0 Å and R1 ~3.8 Å, and the corresponding average θ*-*angles were ~65° and ~67.5°, respectively.

**Figure 3:**
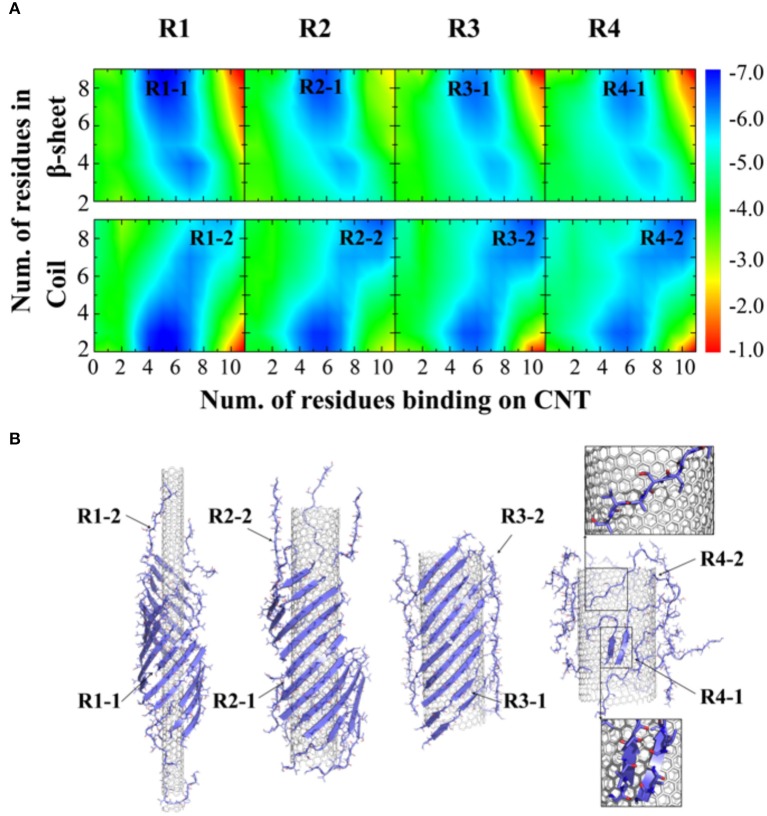
Thermodynamics analysis of NACore assembly in the presence of CNTs of different radii. **(A)** The two-dimensional potential of mean force as a function of the number of residues binding CNT and the number of residues adopt β-sheet (top) or coil (lower) per peptide. CNT radii increased s from R1 to R4. **(B)** Typical snapshots from corresponding simulations illustrated the loss of β-sheet super-coil structures and the transition from β-sheet to coil as CNT radius increases. Peptides corresponding to different free energy basin in **(A)** were highlighted by arrows.

To further understand the thermodynamics of peptide self-assembly on the surface of CNTs, we computed the two-dimensional potential of mean force (2D-PMF)—i.e., the self-assembly free energy landscape—as the function of the number of NACore_68−78_ residues binding with CNT and also the number of residues in β-sheet or coil conformations (Figure 3A). All the 400 ns trajectories from 50 independent simulations were used to capture the early steps of CNT adsorption and conformational conversion. In each case of different radii CNTs, two energy basins were identified—where a β-sheet rich basin (denoted as R1-1, R2-1, R3-1, and R4-1 in Figure 3A with ~6–9 out of 11 residues adopting β-sheet conformations) had only a relatively small number of residues binding CNT (~4–6 out of 11 total residues), and a coil rich basin (labeled as R1-2, R2-2, R3-2, and R4-2 in Figure 3A) with a large number of residues binding CNT (~7–11 out of 11 total residues). In typical self-assembled structures with CNTs of various radii (Figure 3B), peptides corresponding to each of the two energy basins were highlighted. Peptides either formed β-sheets via inter-peptide hydrogen bonding but allowing at most half of the residues binding CNTs (e.g., top inset in Figure 3B), or adopted coil conformations with all residues capable to interact with CNTs (e.g., bottom inset in Figure 3B). With increasing CNT radii, NACore_68−78_ preferred to interact with the flat CNT surface and adopt isolated coil conformation with high translational entropy. The transition from β-sheet rich configurations to coils accompanied the loss of β-sheet supercoils (Figure 2B).

### Peptides form β-Sheets Either Laterally or Vertically on the Surface of Flat Graphene Nanosheets

Examination of coil conformations of NACore_68−78_ on the relatively flat surface of CNT with large radii (Figure 3B) indicated that these peptides tended to bind the CNT in a *straddle* conformation, where all side-chains were in contact with the flattened NP surface. Although classified as the coil conformation because of the lack of backbone hydrogen bonds, the corresponding peptide conformations were rather ordered and highly extended, which were stabilized by extensive side-chain interaction with the CNT surface. Backbone hydrogen bond donors and acceptors were exposed and sticking out of the NP surface. With excessive peptides with respect to the available surface area, incoming peptides might form β-sheets with those backbone-exposed peptides on the NP surface and nucleate the growth of fibril-like β-sheets perpendicular to the NP surface.

**Figure 4:**
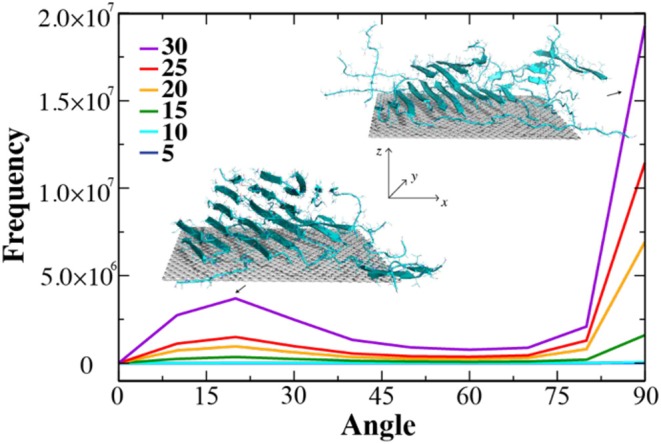
The growth of β-sheets by increasing of number peptides on the surface of graphene nano-sheet. The distribution of angle between graphene surface norm and elongation vectors of neighbor β-sheets were computed from consecutive simulations where the number of NACore peptide were added up to 30 with the increment of 5.

Given the large surface area of the largest radius CNT and the fact that 20 peptides covered only a small fraction of the surface (Figure 2B), the number of peptides required to test the above hypothesis would be high and computationally expensive. Therefore, we chose a graphene nanosheet with a smaller surface area (~3,600 Å^2^ compared to ~8,400 Å^2^ for the largest radius CNT), as the asymptotic case with increasing radius. We monitored the process of NACore_68−78_ self-assembly with increasing number of peptides added to the system in simulations. After initial equilibrium simulations with five peptides in the presence of the graphene nanosheet, we added five new peptides to the simulation system every 50 ns. We stopped the simulations after 30 NACore_68−78_ peptides were introduced into the system in excess of the available graphene surface. As expected, the peptides were able to form β-sheets with the elongation direction either parallel or perpendicular to the surface (e.g., insets of corresponding snapshots in Figure 4). To quantify the directions of β-sheets, we computed the angle (γ) between the graphene norm and local β-sheet elongation directions at each hydrogen-bonded residue pair, defined as the vector connecting Cα atoms of the corresponding two residues. The histograms of γ-angles average over time and independent simulations were computed at different stages (Figure 4). The large angles near 90° corresponded to β-sheets growing collaterally along the graphene surface, while the small angles near 0° were β-sheets with elongation directions along the nanosheet norm. With up to 10 peptides, there were nearly no obvious β-sheets formed on the surface, consistent with previous simulation results that the decreasing CNT curvatures induced peptide in coils (Figures 2, 3). When additional NACore_68−78_ peptides were added, β-sheets started to emerge due to increased inter-peptide interactions. Indeed, there were two peaks around ~90° and ~20°, respectively, confirming the hypothesis of β-sheet growth both collaterally and vertically to the graphene nanosheet surface. Especially with excessive peptides from 25 to 30 total peptides, the increase of the peak around 20° was more than doubled, suggesting the predominant growth of β-sheets vertically. Hence, by tuning CNM surface curvature and the ratio of peptide with respect to the available adsorption surface area, the growth direction of β-sheet rich nanofibers might be controlled and pave the way for the design of high-order hybrid peptide-CNM nanostructures.

## Conclusion

In sum, we applied atomistic DMD simulations to systematically study the self-assembly structure of two types of peptide fragments, Aβ_16−22_ or NACore_68−78_, in the presence of different CNMs in order to determine the physicochemical determinants in terms of different protein sequences, CNT chirality and radii/curvatures. Due to strong interactions between amyloid peptides and CNT, both Aβ_16−22_ and NACore_68−78_ could self-assemble into in-registered β-sheet supercoils on the surface of small radii CNTs. The pitch length of the supercoil is mostly determined by the peptide sequences in terms of the position of hydrophobic or aromatic residues, the CNT radii/curvature, the relative molecular ratio of peptides with respect to the available surface area, and also weakly on the chirality. With increased CNT radii and correspondingly decreased curvature, peptides adsorbed to available CNT surface tended to form coils instead of β-sheets, losing the ability to form β-sheet supercoils. Interestingly, with excessive amyloid peptides with respect to available surface areas of large radii CNTs (e.g., MWCNT) or flat graphene nanosheets, fibrillar aggregates could be nucleated vertically to the NP surface *in silico*. Hence, our study offered a mechanistic insight to peptide self-assembly on CNMs with different peptide sequences and CNM geometries, and should prove valuable for the design of high-order hybrid peptide-CNMs nanostructures.

## Data Availability Statement

All datasets generated for this study are included in the article/Supplementary Material.

## Author Contributions

FD designed the project. YX, YS, and BW conducted atomistic DMD simulations and analyses. YX, YS, and FD wrote the paper. All authors agreed on the presentation of the manuscript.

### Conflict of Interest

The authors declare that the research was conducted in the absence of any commercial or financial relationships that could be construed as a potential conflict of interest.
