# Supplementary material for: Morphological Determinants of Carbon Nanomaterial-Induced Amyloid Peptide Self-Assembly
Source: Front Chem. 2020 Mar 10;8:160. doi: 10.3389/fchem.2020.00160 (PMC7076083; doi:10.3389/fchem.2020.00160)
Supplement: Supplementary file 1 [file Data_Sheet_1.DOCX]

**Supporting Information**

**Uncovering the Morphological Determinants of Carbon Nanomaterial-Induced Amyloid Peptide Self-Assembly**

Yanting Xing,† Yunxiang Sun,† Bo Wang, † and Feng Ding†,*

†Department of Physics and Astronomy, Clemson University, Clemson, SC 29634, USA

Email: fding@clemson.edu

**Supplementary Figure**


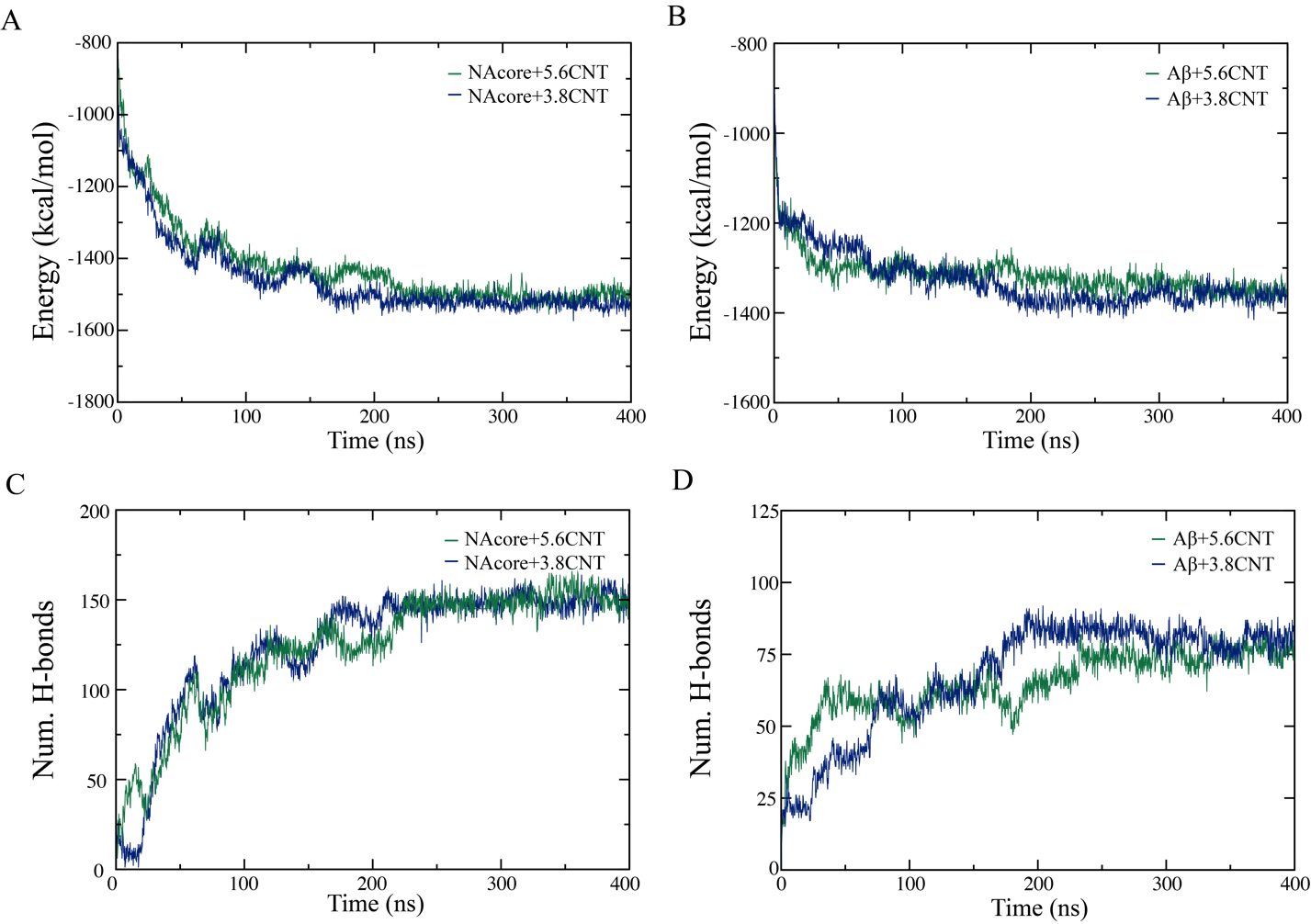


**Figure S1. Typical DMD simulation trajectories in terms of potential energy and number of inter-peptide backbond hydrogen bonds.** For illustrate purpose, we showed one out of 50 independent trajectories of (A)(C) 20 NAcore or (B)(D) 20 Aβ in the presence of different chiral CNTs.


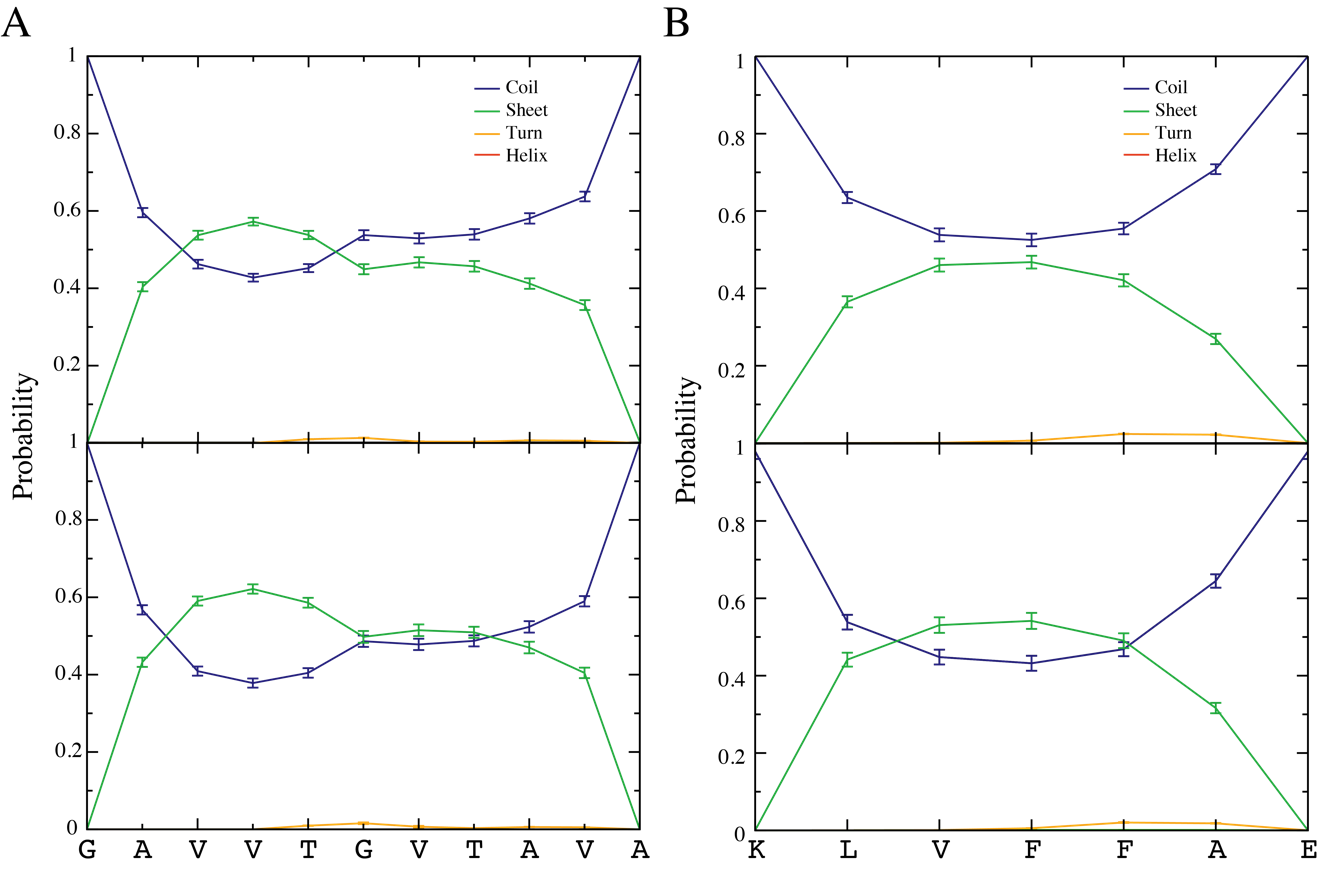


**Figure S2. Secondary structure of NAcore and Aβ in the presence of CNT.** (A) Secondary structure of each NAcore residue in the presence of 3.8 CNT (upper) and 5.6 CNT (lower). (B) Secondary structure of each Aβ residue in the presence of 3.8 CNT (upper) and 5.6 CNT (lower).

**
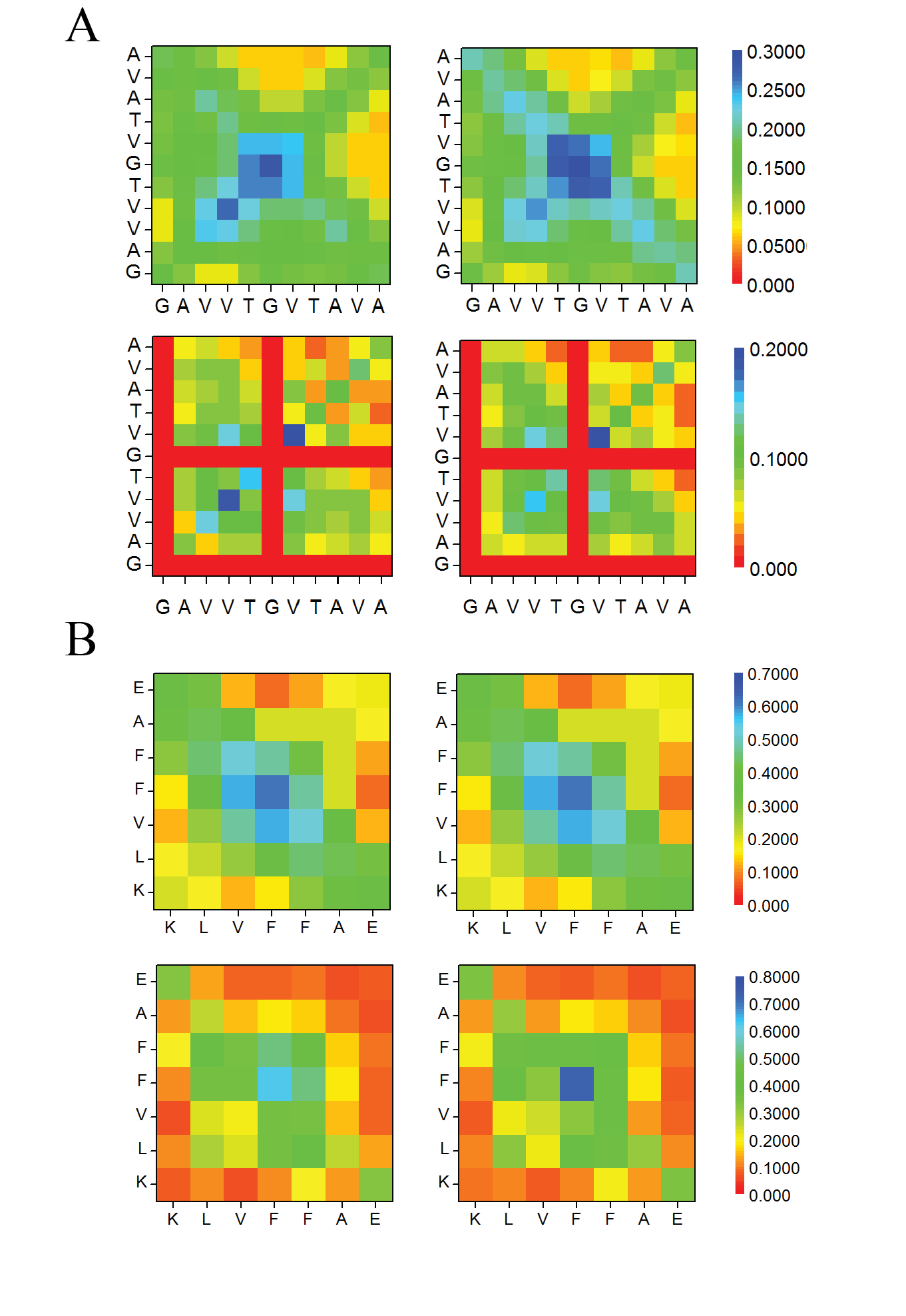
**

**Figure S3. The residue-wise inter-peptide contact frequency maps of NAcore and Aβ in the presence of different chiral CNTs.** (A) Contact frequency map of NAcores’ backbone-backbone (upper) and sidechain-sidechain (lower) in the presence of 3.8 CNT (left) and 5.6 CNT (right). (B) Contact frequency map of Aβs’ backbone-backbone (upper) and sidechain-sidechain (lower) in the presence of 3.8 CNT (left) and 5.6 CNT (right).


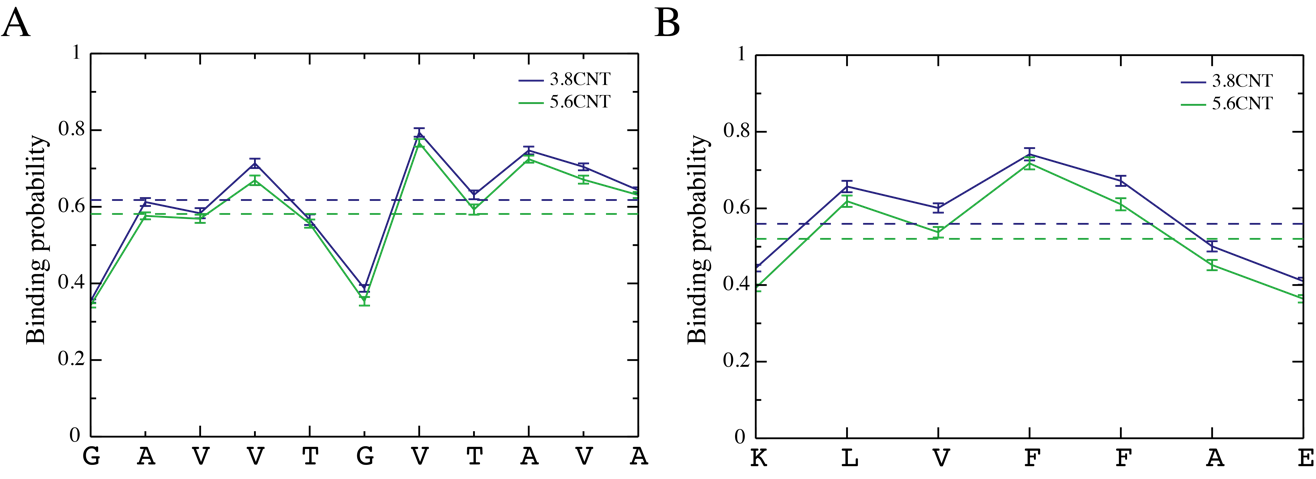


**Figure S4. Binding probability of NAcore and Aβ with CNT.** (A) Binding probability of each NAcore residue with CNT and average binding probability shown in dash lines. (B) Binding probability of each Aβ residue with CNT and average binding probability shown in dash lines.
